# Ion uptake of marigold under saline growth conditions

**DOI:** 10.1186/s40064-016-1815-3

**Published:** 2016-02-20

**Authors:** Nezihe Koksal, Ayfer Alkan-Torun, Ilknur Kulahlioglu, Ebru Ertargin, Eylul Karalar

**Affiliations:** Department of Horticulture, Faculty of Agriculture, University of Cukurova, Balcali, 01330 Adana, Turkey; Department of Soil Science and Plant Nutrition, Faculty of Agriculture, University of Cukurova, Balcali, 01330 Adana, Turkey; Department of Horticulture, Faculty of Agriculture, University of Mustafa Kemal, 31040 Hatay, Turkey

**Keywords:** Bedding plant, Micronutrient and macronutrient, Salt stress, *Tagetes erecta*

## Abstract

Salinity is one of most significant environmental stresses. Marigold is moderately tolerant to salinity stress. Therefore, in this study, the fresh weights of roots and shoots, root_FW_/shoot_FW_ ratio, moisture content of shoots, micronutrient and macronutrient concentrations and ratios of K^+^/Na^+^ and Ca^2+^/Na^+^ in the roots and shoots of marigold were determined under salinity stress. Five salinity treatments (0, 50, 100, 150, and 200 mM NaCl) were maintained. In the current study, salinity affected the biomass of marigold. An increase of more than 100 mM in salt concentrations significantly reduced the shoot fresh weight. Increasing salinity stress increased the ratios of root_FW_/shoot_FW_, which were more significant under high salt levels (150 and 200 mM NaCl). Wet basis moisture contents of the shoots were reduced when salinity stress increased above 100 mM. In this study, salinity stress affected micronutrient and macronutrient uptake. Increases in the salt concentration and decreases in the concentration of Cu^2+^ and Zn^2+^ in the roots and Mn^2+^ and Fe^2+^ in the shoots were significant. Based on an increase in salinity stress, while the Ca^2+^, Mg^2+^, and Na^+^ concentrations increased, the K^+^ concentration decreased in the roots and shoots. Moreover, the K^+^/Na^+^ and Ca^2+^/Na^+^ ratios of the roots and shoots were significantly lower than those of the control in all of the salinity treatments. As a result, under increasing salinity stress, the Ca^2+^, Mg^2+^, K^+^, and Na^+^ uptakes in marigold were significant, revealing the effects of stress.

## Background

Salinity is one of the most important plant-growth-limiting environmental factors. Salinity in soil and/or irrigation waters leads to significant decreases in plant growth. Salinity is increasingly becoming an important concept in terms of environmental planning. In cities where water shortages are common, an increase in green areas has motivated the use of alternative water sources for irrigation. Alternative water resources typically contain a large amount of salt (Navarro et al. 2008; Niu and Rodriguez 2006). Thus, salinity has emerged as a major problem in coastal gardens and landscape planning (Ferrante et al. 2011). The life of some plants is threatened by salinity in coastal areas (Parida et al. 2002).

The impact of salinity on plants may vary depending on the developmental stage and tolerance level of the plant. The effect of salinity stress arises as a result of the combination of the relationship between the morphological, physiological, and biochemical processes of plants (Parida and Das 2005). Plants take in nutrients through the root system. Ion regulation is important under normal conditions and is also vital under saline conditions for plant growth (Aşık et al. 2009). Salinity leads to significant changes in water potential, ion uptake, ion imbalance, ion toxicity and oxidative stress (Grattan and Grieve 1999; Parida and Das 2005). Under salinity stress, changes in the nutritional balance of NaCl result in higher levels of Na^+^/Ca^2+^, Na^+^/K^+^, Na^+^/Mg^2+^, Cl^−^/NO_3_^−^ and Cl^−^/H_2_PO_4_^−^, thus causing plant growth retardation (Grattan and Grieve 1999). Sodium and Cl^−^ can influence the uptake of nutrients by competing with nutrients or affecting the ion permeability of membrane. In most plants, an increase in NaCl in the plant leads to an increase in Na^+^ and Cl^−^ ions but may result in a decrease in N, P, K^+^, and Ca^2+^ (Kandeel et al. 1999; Karimi et al. 2005; Tuna et al. 2007; Navarro et al. 2008). In addition, under saline conditions, increases in the amount of Na^+^, Ca^2+^, Mg^2+^, Cl^−^, SO_4_^2–^, and HCO_3_^−^ in plants also cause toxicity (Valdez-Aguilar et al. 2009a).

Marigold is a significant ornamental plant belonging to the family Compositae that is commonly used in environmental planning and evaluated as a cut flower (Riaz et al. 2013). Among ornamental bedding plants, marigold is known to grow well under saline conditions (Escalona et al. 2012). Some marigold cultivars that are used as cut flowers or as bedding plants in landscaping can be grown by maintaining the quality of plants under saline conditions with an EC_w_ of <8 dS m^−1^ (Valdez-Aguilar et al. 2009a).

Under various marginal conditions, the ability of plants to survive is the main reason for their growth. The reactions of many plants under saline conditions have been reported in several studies (Navarro et al. 2008; Parida et al. 2002; Rodríguez et al. 2005; Romero-Aranda et al. 2001). The salinity tolerance of many ornamental plants that are used in landscaping is not known. In areas with salinity problems, there is not enough information for environmental designers and growers of ornamental plants to recommend the appropriate plant species. In this study, the effects of salinity stress on marigold (*Tagetes erecta* L. ‘Sumo orange’) plants were investigated. For this purpose, the fresh weights, root_FW_/shoot_FW_ ratios, moisture content of shoots, ion concentrations and ratios of K^+^/Na^+^ and Ca^2+^/Na^+^ were determined under saline conditions.

## Methods

### Plant material, growth conditions and salinity treatments

This study was conducted in greenhouses at the Department of Horticulture and the Department of Soil Science and Plant Nutrition, Faculty of Agriculture, University of Cukurova in Adana/Turkey. *Tagetes erecta* L. ‘Sumo orange’ was used as the plant material in the pot experiment. Plant seeds were germinated in peat medium at 22 °C in the dark. After germination, the seedlings were transferred and grown in moist peat. Then, uniformly sized seedlings were transplanted into 2 L plastic pots containing washed peat-perlite (2:1) medium. Each pot was irrigated individually and manually until the harvest as to keep the soil moisture levels around field capacity. The Hoagland solution was applied to each pot as fertilizers. Seedlings were allowed to establish for 2 weeks before treatment. To avoid salt shock in the plants that were exposed to salt treatment, all of the plants except for the control groups were watered with 25 mM NaCl for 15 days at two-day intervals. Five salinity treatments (0, 50, 100, 150, and 200 mM NaCl) were maintained for 25 days at 2-days intervals. The pots were arranged in a complete randomized design (CRD) with three replications, and each replication included seven plants.

### Plant biomass

After harvesting, the plants were uprooted carefully and washed thoroughly in running tap water to remove substrate particles. After rinsing with deionized water, the plants were separated into roots and shoots. Samples were weighed using a digital top-loading weighing balance (Sartorius TE 001) to determine the fresh weight (grams per plant). The root/shoot ratio of fresh weight was also estimated. Later, the plant parts (roots and shoots) were dried to a constant weight, placed in labeled paper bags and oven-dried at 70 °C for 4 days.

The dried shoot samples were weighed to determine the moisture contents. The dried samples of roots and shoots were also used to analyze the ion concentrations.

### Moisture content of the shoots

The moisture content of the samples was determined according to the wetness of the shoots. The wet basis moisture content is the amount of water per unit mass of wet sample and was determined using the following equation:

$$MC_{wb} = \, \left( {{{m{\text{H}}_{2} {\text{O}}} \mathord{\left/ {\vphantom {{m{\text{H}}_{2} {\text{O}}} {m_{fw} }}} \right. \kern-0pt} {m_{fw} }}} \right) \times 100$$

where *MC*_*wb*_ = moisture content on wet basis, *m*H_2_O = mass of water (kg, lb), *m*_*fw*_ = total mass of wet sample (kg, lb).

#### Ion concentration analysis

The dried samples of roots and shoots were used to analyze the ion concentrations. The dry materials were ground and were digested via the dry digestion method. The concentrations of copper (Cu^2+^), manganese (Mn^2+^), iron (Fe^2+^), zinc (Zn^2+^), calcium (Ca^2+^), magnesium (Mg^2+^), potassium (K^+^), phosphorus (P) and sodium (Na^+^) were determined by inductively coupled plasma-atomic emission spectrometry (ICP-AES) (Torun et al. 2013). After determining the ion concentrations, the K^+^/Na^+^ and Ca^2+^/Na^+^ ratios were calculated.

### Statistical analysis

Data were subjected to ANOVA, and the means were separated using the LSD multiple range test at *P* ≤ 0.05. All of the statistical analyses were performed using the JMP 8 software package.

## Results and discussion

### Plant biomass

Fresh weight (FW) changes related to the roots and shoots of marigold under salinity stress are presented in Table 1. The lowest value of root-FW was found at 100 mM NaCl; the other NaCl treatments had no effect on the root-FW. Salinity stress affected the shoot-FW of marigold. Under salinity stress, the highest shoot fresh weights were in the control (17.767 g plant^−1^) and 50 mM (18.620 g plant^−1^) NaCl treatments. However, with NaCl concentrations of more than 100 mM, the shoot-FW decreased by approximately 58 % compared to the control.

**Table 1:** Fresh weights (FW) of the roots and shoots of marigold under salinity stress

NaCl (mM)	Root-FW (g plant^−1^)	Shoot-FW (g plant^−1^)
0	0.829 ± 0.199^a^	17.767 ± 2.031^a^
50	1.038 ± 0.269^a^	18.620 ± 2.957^a^
100	0.510 ± 0.122^b^	7.505 ± 0.689^b^
150	0.814 ± 0.270^a^	7.456 ± 1.154^b^
200	0.916 ± 0.038^a^	7.529 ± 1.590^b^
Prob > f	0.0252	<0.0001
LSD_5 %_	0.303	2.796

The values are the means of seven replicates

The values in columns followed by different letters are significantly different at *P* ≤ 0.05 (least significant difference test)

**Fig. 1:**
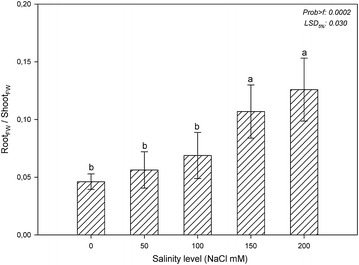
Root-shoot ratio based on the fresh weights of marigold under salinity stress. The *vertical bars* indicate standard deviations, and *different letters* indicate statistically significant differences at *P* ≤ 0.05 (least significant difference test)

A change in the weight of plants due to salinity stress was also observed in the ratio of root_FW_/shoot_FW_ (Fig. 1). In the present study, the ratio of root_FW_/shoot_FW_ increased under salinity stress. Increases in the ratio of root_FW_/shoot_FW_ in the 150 and 200 mM NaCl treatments were significant in comparison to the control and the other treatments. Approximately 2.3- and 2.7-fold increases in the ratios of root_FW_/shoot_FW_ were observed in the 150 and 200 mM NaCl treatment groups compared to the control group.

Growth retardation and fresh and dry weight loss of roots and shoots under salinity stress were revealed in previous studies (Lolaei 2012; Navarro et al. 2008; Villarino and Mattson 2011). In addition, based on the fresh or dry weight, it was demonstrated in several studies that the root/shoot ratios of many plants increase under salinity stress (Debouba et al. 2006; Maggio et al. 2007). Heidari and Akbari (2012) identified 28 and 32 % decreases in the shoot fresh weight of *Tagetes erecta* and *Tagetes tenuifolia* marigold species, respectively, under salinity stress (6 dS m^−1^). Similarly, Villarino and Mattson (2011) determined a decrease in the fresh and dry weights of plants as salinity stress increased (4.0, 7.0, 9.8, 12.1, and 14.2 dS m^−1^) in marigold. Moreover, Rawia et al. (2011) reported that high salinity (3000 mg kg^−1^) decreases the fresh and dry weights of herbs and flowers in marigold. The effects of salinity stress can vary depending on the plant species and variety or the severity of the stress factor. Meanwhile, in a study conducted by Trejo-Téllez et al. (2013), 47 mM NaCl treatment had no effect on the dry weight of leaves and roots in *Tagetes erecta* Linn. Nevertheless, Valdez-Aguilar et al. (2009a) found that the shoot dry weights of two *T. erecta* cultivars decreased at respective rates of 30 and 24 % under low salinity levels (4 dS m^−1^). Within the context of our study, under high salinity stress (≥100 mM NaCl), it is thought that the decreased shoot fresh weight was caused by a failure of the plant to take up water. The results of the shoot moisture content measurements also support this idea.

### Moisture content

**Fig. 2:**
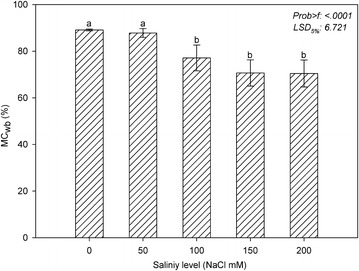
Shoot moisture content of marigold under salinity stress. The *vertical bars* indicate standard deviations, and *different letters* indicate significant differences at *P* ≤ 0.05 (least significant difference test)

The wet-basis moisture content (MC_wb_) of shoots decreased as the salinity stress increased (Fig. 2). The wet-basis moisture contents of shoots in the 0, 50, 100, 150, and 200 mM NaCl treatments were approximately 89, 88, 77, 71, and 70 %, respectively. While there was no significant difference in the wet-basis moisture content of the shoots in the control and 50 mM NaCl treatments, MC_wb_ significantly decreased as the salinity stress increased above 100 mM. In the 100, 150, and 200 mM NaCl treatments, the wet-basis moisture contents of the shoots decreased at the respective rates of 13.5, 20.7, and 20.9 % compared to the control (Fig. 2).

In previous studies, plant water loss under salinity stress was assessed using different parameters that are associated with water uptake. Rodriguez et al. (1997) reported that the leaf water potential and leaf relative water content values of tomato plants that were exposed to salinity stress (100 mM NaCl) were lower than the control values. Similarly, Tuna et al. (2007) reported that treatment with 75 mM NaCl reduced the leaf-RWC values of tomato plants. In addition, in accordance with our results, in a study by Navarro et al. (2008), along with the disturbance of ornamental and other plant characters of *Arbutus unedo* seedlings, the leaf water potential decreased under salinity stress. In agreement with the results of previous studies, it was determined in our study that there is a failure of water uptake in marigold. The wet-basis moisture content of the shoot tissues significantly decreased under high salt concentrations (Fig. 2), indicating a failure of water uptake in marigold under high salinity stress (≥100 mM NaCl).

### Micronutrient and macronutrient concentrations

In this study, salinity stress affected the micronutrient and macronutrient uptake (Tables 2, 3). In Table 2, the Cu^2+^, Mn^2+^, Fe^2+^, and Zn^2+^ concentrations in the roots and shoots of marigold under salinity stress are presented. Under increasing salinity stress (≥100 mM NaCl), a decrease in the Cu^2+^ uptake of root tissues was noted. The change in the Cu^2+^ concentration of shoots was statistically insignificant. The change in the Mn^2+^ concentrations in the roots depending on the increasing salt treatments was not very significant. Moreover, the lowest Mn^2+^ concentration was found in the 100 mM NaCl and 200 mM NaCl treatments. There was no significant difference between the Mn^2+^ concentrations of the shoots in the control and 50 mM NaCl treatments, the latter of which was the lowest salinity stress. However, the Mn^2+^ concentration in the shoots decreased significantly as the salt concentration increased (≥100 mM NaCl). The lowest Mn^2+^ concentration was found in the 100 (~98.29 mg kg^−1^) and 150 (~100.20 mg kg^−1^) mM NaCl treatments. The iron concentration of the roots and shoots showed significant changes under increasing salinity stress. The lowest Fe^2+^ concentration (77.30 mg kg^−1^) in the root occurred at 200 mM NaCl, which was the highest NaCl level. Regarding the shoot tissues, there was no significant difference between the Fe^2+^ concentrations in the control and 50 mM NaCl treatments, while the Fe^2+^ concentration significantly decreased with an increase in the salt concentration. The zinc concentrations of the roots were lower in all of the NaCl treatments than in the control. The zinc concentration in the shoots was not statistically significant.

**Table 2:** Micronutrients concentrations of roots (R) and shoots (S) of marigold under salinity stress

NaCl (mM)	Cu^2+^ (mg kg^−1^)		Mn^2+^ (mg kg^−1^)		Fe^2+^ (mg kg^−1^)		Zn^2+^ (mg kg^−1^)	
	R	S	R	S	R	S	R	S
0	11.68 ± 2.08^a^	11.62 ± 0.39	63.38 ± 8.82^a^	141.46 ± 11.39^a^	137.11 ± 26.62^a^	95.03 ± 4.49^ab^	60.47 ± 12.71^a^	54.72 ± 7.12
50	12.15 ± 1.03^a^	10.81 ± 0.59	59.13 ± 9.61^a^	156.24 ± 7.35^a^	128.90 ± 32.82^a^	110.78 ± 24.16^a^	42.72 ± 11.77^b^	50.37 ± 6.95
100	3.44 ± 0.70^b^	10.41 ± 0.84	37.22 ± 5.41^b^	98.29 ± 14.1^c^	101.53 ± 30.07^ab^	75.27 ± 10.7^b^	26.27 ± 1.82^c^	43.15 ± 0.78
150	3.15 ± 0.19^b^	10.45 ± 1.62	67.07 ± 9.91^a^	106.20 ± 7.67^c^	132.23 ± 33.89^a^	83.42 ± 20.08^b^	45.68 ± 7.85^b^	48.65 ± 6.10
200	5.14 ± 1.84^b^	9.79 ± 0.80	31.52 ± 4.76^b^	122.97 ± 6.65^b^	77.30 ± 11.91^b^	76.94 ± 6.76^b^	21.11 ± 5.73^c^	54.73 ± 9.39
Prob > f	<0.0001	0.1424	<0.0001	<0.0001	0.0419	0.0265	0.0001	0.1364
LSD_5 %_	2.058	ns	12.063	14.883	42.522	23.041	13.446	ns

The values are the means of seven replicates

The values in columns followed by different letters are significantly different at *P* ≤ 0.05 (least significant difference test)

*ns* not significant

**Table 3:** Macronutrients concentrations of roots (R) and shoots (S) of marigold under salinity stress

NaCl (mM)	Ca^2+^ (g kg^−1^)		Mg^2+^ (g kg^−1^)		K^+^ (g kg^−1^)		P (g kg^−1^)		Na^+^ (g kg^−1^)	
	R	S	R	S	R	S	R	S	R	S
0	8.04 ± 0.69^c^ ^y^	2.41 ± 0.18^b^	1.34 ± 0.05^c^	4.12 ± 0.84^c^	4.23 ± 1.07^a^	4.61 ± 0.27^ab^	3.88 ± 0.47^b^	4.22 ± 0.62	10.34 ± 1.22^b^	4.89 ± 1.66^e^
50	10.07 ± 2.03^bc^	2.39 ± 0.07^b^	1.26 ± 0.01^c^	5.19 ± 0.34^c^	2.63 ± 0.33^b^	4.80 ± 0.33^a^	3.69 ± 0.30^b^	4.28 ± 0.91	32.98 ± 4.27^a^	21.76 ± 6.03^d^
100	12.64 ± 2.75^abc^	3.10 ± 0.36^a^	3.00 ± 0.39^b^	24.75 ± 0.35^ab^	1.08 ± 0.11^c^	4.25 ± 0.70^abc^	9.80 ± 2.43^a^	4.03 ± 0.58	31.16 ± 4.04^a^	37.71 ± 4.55^c^
150	15.64 ± 5.82^a^	2.88 ± 0.06^a^	4.64 ± 1.05^a^	26.71 ± 3.37^a^	1.56 ± 0.42^c^	3.76 ± 0.28^c^	9.39 ± 3.13^a^	3.88 ± 0.33	37.49 ± 6.62^a^	51.67 ± 4.14^b^
200	14.62 ± 2.59^ab^	3.01 ± 0.34^a^	3.69 ± 0.93^ab^	21.53 ± 4.38^b^	1.54 ± 0.36^c^	3.92 ± 0.64^bc^	3.48 ± 0.92^b^	4.90 ± 0.93	35.45 ± 5.33^a^	62.27 ± 9.15^a^
Prob > f	0.0257	0.0013	<0.0001	<0.0001	<0.0001	0.0377	0.0001	0.3445	<0.0001	<0.0001
LSD_5 %_	4.894	0.362	0.983	3.781	0.845	0.726	2.766	ns	7.011	8.544

The values are the means of seven replicates

The values in columns followed by different letters are significantly different at *P* ≤ 0.05 (least significant difference test)

*ns* not significant

The effects of salinity stress on microelement uptake have been investigated in various studies (Villora et al. 2000; Lao and Plaza 2013). However, the relationship between salinity and microelement uptake is complex. An increase or decrease may be observed in microelement uptake, or salinity may not have an effect on the microelement concentration of the plant. These differences result from factors such as plant species, plant tissues, level of salinity stress and composition, microelement concentration in the growth medium, growth conditions and stress duration (Grattan and Grieve 1999). In a study by Rahman et al. (1993), Mn^2+^ and Cu^2+^ accumulation in maize shoots under salinity stress decreased. Eom et al. (2007) suggested that salinity stress does not affect the Fe^2+^ or Zn^2+^ uptake of six different types of ground cover plants but reduces the concentration of Cu^2+^. In another study, Valdez-Aguilar et al. (2009b) indicated that increasing salinity in nutrient solution has little effect on the micronutrient uptake of marigold. In the same study, an increase in EC_w_ in the nutrient solution affected marigold varieties differently. Despite the EC_w_ increase, Cu^2+^ and Zn^2+^ accumulation in marigold did not show a pattern consistent with the results of our study. The Mn^2+^ concentration showed a decreasing trend in *T. erecta* varieties. In agreement with similar studies, a decrease in Cu^2+^, Fe^2+^, and Mn^2+^ concentration was observed in our study depending on the increase in salinity stress, while salinity stress has no effect on Zn^2+^ uptake normally.

In Table 3, the Ca^2+^, Mg^2+^, K^+^, P, and Na^+^ concentrations in the roots and shoots of marigold under salinity stress are presented.

Salinity stress affected the Ca^2+^ concentration (Table 3). In general, as the salt concentration increased, the Ca^2+^ concentration of marigold also increased. The highest Ca^2+^ concentration in roots (15.64 g kg^−1^) was observed in the 150 mM NaCl treatment, followed by the 200, 100, 50, and 0 mM NaCl treatments. Regarding the shoot tissues, the Ca^2+^ concentrations in the control and 50 mM NaCl treatments were not significantly different, but the Ca^2+^ concentration significantly increased when NaCl was applied at concentrations of 100 mM and greater. The Ca^2+^ concentration of plants under salinity stress must be high to maintain plant growth (Tuna et al. 2007). Correspondingly, in our study, it was found that growth retardation was not observed due to an increasing NaCl concentration in marigold, which is moderately tolerant to salt (data not shown). This result was accompanied by an increase in the Ca^2+^ concentration of plant tissues. In contrast, at the end of the study, drying was observed in plants under high salt treatments (100 mM and greater).

Along with Ca^2+^, there was an increase in the Mg^2+^ concentrations of root and shoot tissues of marigold as the salt concentration increased (Table 3). The lowest Mg^2+^ concentration in the roots and shoots was found in the control and 50 mM NaCl and treatments, and Mg^2+^ concentration increased in parallel with the increased salinity stress. The highest Mg^2+^ concentration in the roots and shoots was observed in the 150 mM NaCl treatment, followed by the other high-salt treatments (100 and 200 mM NaCl). Similar to our results, Valdez-Aguilar et al. (2009b) determined that the Mg^2+^ concentration of marigold leaves increased in parallel with an EC_w_ increase in the nutrient solution. Likewise, Carter et al. (2005) stated that the Mg^2+^ concentration of *Celosia argentea* increased due to the increased salinity stress.

Under salinity stress, changes in the K^+^ concentrations of the roots and shoots were significant (Table 3). The K^+^ concentration in the roots and shoots decreased depending on the increase in salinity stress. The potassium concentration in roots was found to be lower in all NaCl treatments compared to the control. The potassium concentration in the roots reduced 1.6, 3.9, 2.7, and 2.7 times, respectively, in the 50, 100, 150, and 200 mM treatments compared to the control. The K^+^ concentration of the shoots decreased approximately 1.1, 1.2, and 1.2 times in the 100, 150, and 200 mM NaCl treatments, respectively, compared to the control. Our results agree with those in the literature. An increase in Na^+^ uptake and a decrease in K^+^ uptake under salinity stress have been shown in various studies (Debouba et al. 2006; Karimi et al. 2005; Lolaei 2012; Tuna et al. 2007; Navarro et al. 2008). Under saline conditions, Na^+^ inhibits K^+^ uptake by competing with K^+^ ions, which are similar in terms of ionic diameter and electrical load (Grattan and Grieve 1999). In addition, the negative relationship between Mg^2+^ and K^+^ has also been reported by many researchers (Carter et al. 2005; Valdez-Aguilar et al. 2009b). In our study, there was an increase in Mg^2+^ in the shoots and roots despite a decrease in K^+^.

Under increasing salinity stress, changes in the P concentration in the roots of marigolds were statistically significant, whereas P concentration in the shoots was insignificant (Table 3). The highest P concentration in roots occurred in the 100 mM NaCl (9.80 g kg^−1^) and 150 mM NaCl (9.39 g kg^−1^) treatments. The P uptake of plants under salinity stress is complex. Under salinity stress, P concentrations in many different plant species decrease, increase or remain unchanged. Factors such as habitat, plant species and variety, plant growth stage, level and composition of salt stress, and P concentration in the growth medium may lead to differences in P uptake (Grattan and Grieve 1994).

As the salinity stress increased, the change in the Na^+^ concentration of plant tissues was statistically significant (Table 3). Sodium concentration in the roots was higher in all of the salinity treatments than in the control. However, the Na^+^ concentration in shoots increased depending on the increase in salinity stress. The sodium concentration of the shoots increased approximately 5, 8, 11, and 13 times in the 50, 100, 150, and 200 mM treatments, respectively, compared to the control. Valdez-Aguilar et al. (2009b) reported that the Na^+^ concentration in marigold under salinity stress was lower compared to that of other ornamental plants. In addition, the same researchers reported an effective mechanism limiting the transport of Na^+^ from the roots to the leaves. In our study, there was no significant limitation regarding the Na^+^ transport of the plant. In contrast, in the 50 mM NaCl treatment, which was the lowest level of salinity stress, Na^+^ was high levels in the roots. This result shows that Na^+^ transport in marigold can be limited under low NaCl concentrations. Generally, to tolerate saline conditions with more Ca^+2^ or K^+^ ion uptake, plants limit Na^+^ uptake, thus providing an ion balance (Hussain et al. 2008). In our study, the increase in Na^+^ and Ca^+2^ concentrations agrees with the increase in salinity stress, and the results agree with those in the literature.

**Fig. 3:**
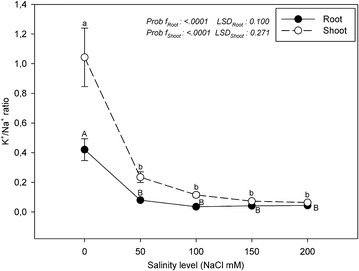
K^+^/Na^+^ ratios of the roots and shoots of marigold under salinity stress

**Fig. 4:**
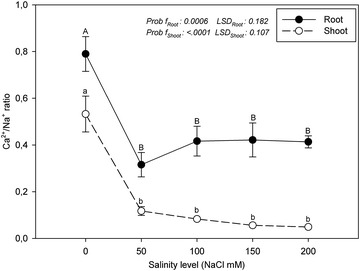
Ca^+2^/Na^+^ ratios of the roots and shoots of marigold under salinity stress

The effect of salinity stress on the K^+^ and Ca^2+^ uptake of marigold is more clearly understood than the ratios of K^+^/Na^+^ and Ca^2+^/Na^+^ (Figs. 3, 4). Under saline conditions, the K^+^/Na^+^ and Ca^2+^/Na^+^ ratios are significant indicators in terms of the salt tolerance or damage of the plants (Lopez and Satti 1996; Navarro et al. 2008). Under saline conditions, a decrease in the Ca^2+^/Na^+^ ratio causes the deterioration of membrane permeability and the excessive uptake of the other salt ions, especially Na^+^ (Villora et al. 2000). Tuna et al. (2007) stated that the K^+^/Na^+^ and Ca^2+^/Na^+^ ratios of tomato plants under salinity stress are lower than those in the control group. In agreement with this result, in our study, the K^+^/Na^+^ and Ca^2+^/Na^+^ ratios of the roots and shoots were significantly lower than in the control in all of the salinity treatments.

## Conclusions

In the current study, salt concentrations greater than 100 mM NaCl significantly decreased the weights of the shoots, while high salt levels (150 and 200 mM NaCl) significantly increased the root_FW_/shoot_FW_ ratio. There was a failure in water uptake when NaCl was applied at a concentration >100 mM. As a consequence of the study, salinity stress was determined to have a significant impact in terms of micronutrient and macronutrient uptake. The decrease in Cu^2+^ and Zn^2+^ of roots has been considered. It is remarkable that there is a decrease in the Mn^2+^ and Fe^2+^ of shoots. While the Ca^2+^, Mg^2+^, and Na^+^ increased in the roots and shoots as the salinity stress increased, the K^+^ concentration decreased. Phosphorus concentration increased at high salt levels (100 and 150 mM) in the roots. Despite the increasing salinity stress, an increase in Ca^2+^ and Mg^2+^ leads to an increase in the plant’s tolerance to stress. Under saline conditions, the Ca^2+^, Mg^2+^, K^+^, and Na^+^ uptakes of marigold were important parameters in terms of revealing the effects of stress.
